# 4442 CTSA Recruitment Resources: An Inventory of What CTSA Hubs Are Currently Offering

**DOI:** 10.1017/cts.2020.379

**Published:** 2020-07-29

**Authors:** Brenda Hudson, Nyiramugisha Niyibizi, Scott McIntosh, Ashley Sipocz, Emily Paku, Laurie A. Lebo, Carrie Dykes

**Affiliations:** 1 Indiana Clinical and Translational Sciences Institute; 2 Emory University, Georgia Clinical and Translational Science; 3 University of Rochester Medical Center; 4 Northwestern University; 5 Georgetown-Howard Universities CTSA; 6 Vanderbilt University Medical Center; 7 Univ of Rochester, Clinical & Translation Science Institute

## Abstract

OBJECTIVES/GOALS: The objective of this project was to determine the research volunteer recruitment capabilities and methodologies currently utilized by CTSA Hubs in order to disseminate recruitment best practices and create collaborations across institutions. METHODS/STUDY POPULATION: The CTSA Recruitment and Retention working group developed a REDCap survey to collect information about what participant recruitment and retention resources and processes are being used at CTSA institutions to support investigators. It was distributed to CTSAs between May and July 2019. The survey, consisting of over 50 multiple choice and short answer questions, is an updated version of a 2016 survey. Institutions reported on registry use, feasibility assessment use, clinical trial listings, experience recruiting special populations, program operations and evaluation, workforce education, social media use and other recruitment resources. RESULTS/ANTICIPATED RESULTS: 40 of the 64 CTSA institutions completed the survey. Almost all of those responding are providing investigators access to a registry either favoring their institutional registry (45%) or ResearchMatch (34%) with most (85%) leveraging ResearchMatch to some extent. Over 80% of the CTSAs are providing investigators recruitment consultations, feasibility assessments, study listings, and EMR Utilization. 73% of the CTSAs are assisting with study materials with 47% offering social media assistance. Additionally, over half the respondents indicated they were successful recruiting healthy volunteers, patients, seniors, pregnant women, minors, low-income populations and underrepresented minorities; however, most found recruiting non or limited English speaking persons and rural populations challenging. DISCUSSION/SIGNIFICANCE OF IMPACT: A variety of recruitment and retention resources exist across CTSA institutions, and this inventory serves as a way to compile these services and foster collaboration across institutions. Additionally, it allows CTSAs to identify services not currently being offered that could improve outcomes while also creating opportunities for collaboration.

